# S91. CLINICAL CHARACTERISTICS OF FORMAL THOUGHT DISORDER IN SCHIZOPHRENIA

**DOI:** 10.1093/schbul/sby018.878

**Published:** 2018-04-01

**Authors:** Joonho Choi, Han-Yong Jung, Joo Eon Park, Hoseon Lee, Seon-Cheol Park

**Affiliations:** 1 Hanyang University Guri Hospital; 2 Soonchunhyang University Bucheon Hospital; 3 Keyo Hospital; 4 St. Andrew Hospital; 5 Inje University College of Medicine and Haeundae Paik Hospital

## Abstract

**Background:**

Our study aimed to present the distinctive correlates of formal thought disorder in patients with schizophrenia, using the Clinical Language Disorder Rating Scale (CLANG).

**Methods:**

We compared the formal thought disorder and other clinical characteristics between schizophrenia patients with (n = 82) and without (n = 80) formal thought disorder. Psychometric scales including the CLANG, Brief Psychiatric Rating Scale (BPRS), Young Mania Rating Scale (YMRS), Calgery Depression Scale for Schizophrenia (CDSS) and Word Fluency Test (WFT) were used.

**Results:**

After adjusting the effects of age, sex and total scores on the BPRS, YMRS and WFT, the subjects with disorganized speech presented significantly higher score on the poverty of contents of abnormal syntax (F = 7.08, P = 0.01), lack of semantic association (F = 8.02, P =0.01), disclosure failure (F = 60.97, P < 0.001), pragmatics disorder (F = 11.94, P = 0.01), dysarthria (F = 13.61, P < 0.001), and paraphasic error (F = 8.25, P = 0.01) items than those without formal thought disorder. With defining the mentioned item scores as covariates, binary logistic regression model predicted that disclosure failure (adjusted odds ratio [aOR] = 5.88, P < 0.001) and pragmatics disorder (aOR = 2.17, P = 0.04) were distinctive correlates of formal thought disorder in patients with schizophrenia.

**Discussion:**

Disclosure failure and pragmatics disorder might be used as the distinctive indexes for formal thought disorder in patients with schizophrenia.

